# Urinary Volatile Organic Compounds as Non-Invasive Biomarkers to Distinguish Hepatocellular Carcinoma from Cirrhosis: A Multicentre Diagnostic Accuracy Study

**DOI:** 10.3390/s26185907

**Published:** 2026-09-18

**Authors:** Yaqza Hussain, Arif Atique, Ayman Bannaga, Krishna C. Persaud, Ramesh P. Arasaradnam, Tendency 2 Study Group

**Affiliations:** 1 Department of Gastroenterology, Sandwell and West Birmingham NHS Trust, Birmingham B71 4HJ, UK; yaqza.hussain4@nhs.net; 2 Institute of Precision Diagnostics & Translational Medicine, University Hospitals Coventry and Warwickshire NHS Trust, Coventry CV2 2DX, UK; arif.atique@nhs.net; 3 Warwick Medical School, University of Warwick, Coventry CV4 7AL, UK; 4 Department of Gastroenterology, Queen Elizabeth Hospital Birmingham, Birmingham B15 2GW, UK; ayman.bannaga@uhb.nhs.uk; 5 Department of Chemical Engineering and Analytical Science, University of Manchester, Manchester M13 9PL, UK; krishna.persaud@manchester.ac.uk

**Keywords:** hepatocellular carcinoma, cirrhosis, volatile organic compounds, biomarkers, early diagnosis, non-invasive screening

## Abstract

Hepatocellular carcinoma (HCC) typically arises on a background of cirrhosis and carries high mortality, in large part because current surveillance tools—ultrasound and serum AFP—have suboptimal sensitivity for early-stage disease. We investigated whether urinary volatile organic compounds (VOCs) could provide a more accurate non-invasive approach. Across four UK centres, 256 participants were recruited: 72 with confirmed HCC and 184 with cirrhosis but no malignancy. Complete urine VOC profiles were obtained using an eight-sensor electronic nose in 190 participants (58 HCC, 132 cirrhosis). Seven of the eight sensors showed statistically significant differences between groups, all reaching *p* < 0.001 (S1–S4, S6–S8), while one (S5) did not reach significance (*p* = 0.682). Principal components analysis demonstrated clear separation between HCC and cirrhosis populations, with the first two components accounting for 70.7% of total variance. A radial basis function neural network, trained on 20% of the data and tested on an independent second replicate, achieved sensitivity of 98.28% (95% CI: 90.76% to 99.96%), specificity of 96.97% (95% CI: 92.42% to 99.17%), PPV of 93.4% (95% CI: 84.44% to 97.40%) and NPV of 99.2% (95% CI: 94.83% to 99.89%). The area under the ROC curve was 0.99 (95% CI: 0.959–1.000). Performance was robust in a mixed treated/untreated cohort, with 50% of HCC patients untreated at sampling; two treated cases fell within the cirrhosis cluster, indicating that VOC profiling may be most sensitive for active, untreated disease. **What’s New?** Urinary VOC profiling demonstrates improved diagnostic accuracy for distinguishing HCC from cirrhosis compared to conventional screening of serum alpha-fetoprotein combined with ultrasound imaging. This non-invasive approach offers substantial clinical potential for HCC screening in high-risk cirrhotic populations at lower cost but will need validation and economic evaluation in larger independent cohorts.

## 1. Introduction

Hepatocellular carcinoma (HCC) ranks sixth among the most common cancers globally and third for cancer deaths, affecting more than 900,000 people each year [1]. The disease predominantly affects patients with established liver disease, with cirrhosis present in 80–90% of cases. The principal causes of progression from cirrhosis to cancer are chronic hepatitis B and C infections, alcohol-related liver disease, and increasingly, fatty liver disease (both NAFLD and the newly termed MASLD) [2].

The annual incidence of HCC in cirrhotic populations is 2 to 6% [3], with a multicentre US surveillance study reporting 2.4% annual incidence regardless of aetiology [4]. Despite this well characterised risk, five year HCC survival remains below 18% globally [1], driven primarily by late stage diagnosis.

The clinical staging and management of hepatocellular carcinoma (HCC) are largely determined by the Barcelona Clinic Liver Cancer (BCLC) classification system, which stratifies patients into five prognostic categories based on tumour burden, hepatic function, and performance status. This framework provides the principal basis for selecting therapeutic strategies and estimating survival outcomes. Curative modalities that include surgical resection, liver transplantation, and percutaneous ablation are primarily indicated for patients with BCLC stage 0 or A disease, in whom five-year survival rates may exceed 70%. In contrast, individuals presenting with advanced (BCLC stage C) or terminal disease (BCLC stage D) have substantially poorer prognoses, with median survival of approximately 4–8 months and 3 months, respectively [5].

The marked difference in outcomes between early and late-stage detection warrants effective surveillance strategies. Current international guidelines recommend six-monthly surveillance for high-risk populations, including patients with cirrhosis (regardless of aetiology), chronic hepatitis B patients, and patients with advanced fibrosis secondary to chronic hepatitis C infection [6].

Despite widespread implementation, current surveillance modalities for hepatocellular carcinoma (HCC) have substantial performance limitations. Ultrasound, the principal imaging tool in routine surveillance, exhibits a pooled sensitivity of only ~45% in real-world practice and performs particularly poorly in identifying early-stage tumours smaller than 2 cm [7]. Detection is further hindered by the heterogeneous echotexture characteristic of cirrhotic liver parenchyma, while operator-dependent variability introduces additional inconsistency in diagnostic accuracy. Serum AFP, while routinely incorporated into HCC surveillance algorithms, demonstrates inadequate diagnostic sensitivity of approximately 61% across thresholds of 20–100 ng/mL, with a specificity of 86% [8].

These limitations in existing surveillance methodologies have prompted intensive research into novel biomarker platforms. Volatile organic compounds (VOCs) represent metabolic by-products released through various biological processes, detectable in bodily fluids and exhaled breath. Recent evidence suggests that disease-specific metabolic alterations generate unique VOC signatures that may serve as diagnostic biomarkers. VOCs are hypothesised to originate from cytochrome P450-mediated metabolism, with distinct profiles emerging in malignant conditions [9].

Preliminary investigations have demonstrated promising results for VOC-based HCC detection. Sukaram et al. identified exhaled acetone dimer as significantly different in HCC compared to cirrhosis and chronic hepatitis B [10], whereas Nazir et al. demonstrated that gas chromatography–mass spectrometry coupled with machine learning could differentiate HCC from healthy controls [11].

The TENDENCY study, a liver biomarker programme initiated at University Hospitals Coventry and Warwickshire by Arasaradnam and Bannaga, conducted a pilot phase urinary VOC analysis using gas chromatography time of flight mass spectrometry and identified seven compounds provisionally associated with HCC including 4-methyl-2,4-bis(p-hydroxyphenyl)pent-1-ene, 2-butanone, and 2-hexanone [12]. The pilot phase enrolled 20 HCC cases compared against healthy volunteers and MASLD patients rather than established cirrhosis controls, the population in whom surveillance is clinically indicated and among whom HCC discrimination is most challenging. Compound identification relied on gas chromatography ion mobility spectrometry and time of flight mass spectrometry, techniques that, whilst analytically powerful, are not suited to point-of-care deployment.

This present study (Tendency 2) sought to address some of these limitations and was therefore designed as a prospective validation for HCC detection using an established cirrhosis group as a comparator across four NHS centres. Further, instead of a high-end laboratory-based mass spectrometry approach, this study utilised a deployable eight sensor electronic nose platform capable of point-of-care application.

## 2. Materials and Methods

### 2.1. Study Design and Setting

This prospective multicentre diagnostic accuracy study was conducted across four UK National Health Service (NHS) medical centres: University Hospitals Coventry and Warwickshire NHS Trust (UHCW), Royal Stoke University Hospital, New Cross Hospital, and Russells Hall Hospital (RHH). The study received ethical approval from Northeast-York Ethics Committee (approval number: 19/NE/0213). The Integrated Research Application System (IRAS) number for the study is 260179, and the National Institute for Health and Care Research (NIHR) portfolio ID is 42438. All participants provided written informed consent prior to enrolment. The study was conducted in accordance with the Declaration of Helsinki and adhered to Good Clinical Practice guidelines.

### 2.2. Sample Size Calculation

Sample size was calculated using Buderer’s method for diagnostic accuracy studies, based on pilot data from the TENDENCY study [13]. The sensitivity (89%) and specificity (81%) assumptions were derived from TENDENCY pilot findings for plasma methylated Septin 9 (mSEPT9), representing the best available preliminary performance data for this study population at the time of design. An HCC prevalence of 27% in the target cirrhotic surveillance population was assumed, with 95% confidence intervals and a z-value of 1.96. The following formulae were applied:

N_1_ = (1.96^2^ × Sensitivity × (1 − Sensitivity))/(0.10^2^ × Prevalence)

N_2_ = (1.96^2^ × Specificity × (1 − Specificity))/(0.10^2^ × (1 − Prevalence))

These calculations yielded a required total sample size of 300 participants (80 HCC cases and 220 cirrhosis controls; 1:2.75 ratio), corresponding to a precision of ±6.8% around the sensitivity estimate and ±5.2% around the specificity estimate. No dropout rate was assumed, as sample collection was a single time-point procedure.

**Figure 1:**
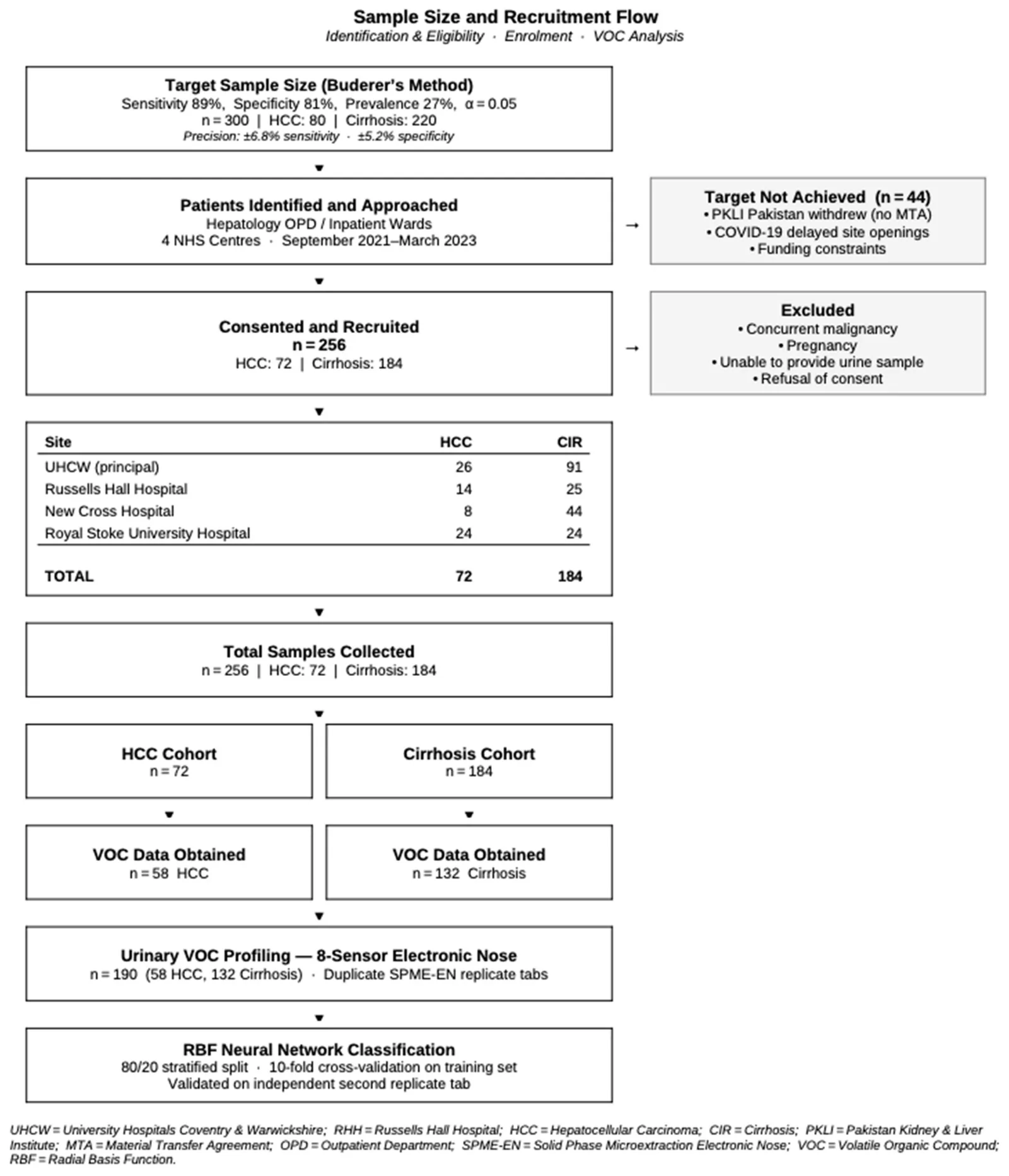
Sample size calculation and participant recruitment flow for the multicentre urinary volatile organic compound (VOC) diagnostic accuracy study.

The study enrolled 256 participants (72 HCC, 184 cirrhosis), of whom 190 provided evaluable VOC data, representing 85% of the target sample size; the sensitivity estimates were only marginally increased without significant implications on diagnostic accuracy (Figure 1).

### 2.3. Participant Selection and Recruitment

Consecutive eligible patients were recruited from hepatology outpatient clinics and inpatient wards between September 2021 and March 2023.

**Inclusion criteria—HCC cohort:** (1) histologically or radiologically confirmed HCC according to EASL-EORTC criteria; (2) age ≥ 18 years; (3) ability to provide informed consent.

**Inclusion criteria—Cirrhosis cohort:** (1) confirmed cirrhosis diagnosis by histology, radiological features, or clinical assessment with laboratory and imaging findings consistent with portal hypertension; (2) absence of HCC on contemporaneous imaging; (3) age ≥ 18 years; (4) ability to provide informed consent.

**Exclusion criteria (both cohorts):** (1) concurrent malignancy; (2) pregnancy; (3) inability to provide urine sample; (4) refusal of consent.

### 2.4. Clinical Data Collection

Comprehensive clinical and demographic data were collected at enrolment using standardised case report forms. Variables recorded included: age, sex, body mass index (BMI), liver disease aetiology, smoking history, alcohol consumption history, comorbidities, concurrent medications, recent radiological findings, histopathological confirmation (HCC cohort), HCC treatment modality (if applicable), and available biochemical parameters including AFP, alanine aminotransferase (ALT), alkaline phosphatase (ALP), albumin, international normalised ratio (INR), prothrombin time, and gamma-glutamyl transferase (GGT).

### 2.5. VOC Analysis Methodology

Urinary VOC profiling was conducted using the SENSAM volatile sampling system, as previously described by Bannaga et al. [14]. The system employs polymer-coated tabs that preconcentrate volatile compounds from the headspace above a liquid matrix. These polymers exhibit differing affinities for a broad range of volatile chemicals, enabling efficient trapping of complex headspace mixtures. The approach is conceptually analogous to solid-phase microextraction, a well-established technique for preconcentrating headspace volatiles prior to gas chromatography–mass spectrometry analysis [15]. The amount of analyte extracted is proportional to its concentration in the sample, if equilibrium is achieved.

For each specimen, duplicate tabs were suspended in the sample headspace for 5 min to capture VOCs. After exposure, tabs were removed and sealed in plastic receptacles. Exposed tabs can be stored at room temperature for up to one week without appreciable loss of volatiles, and for extended periods at −80 °C.

Each tab was subsequently introduced into a custom-built electronic nose sensing instrument (SENSAM volatile sampling system; University of Manchester, Manchester, UK) comprising an array of eight conducting-polymer metal oxide gas sensors integrated with a thermal desorption unit. Each sensor responds to a partially overlapping but distinct range of volatile chemicals, enabling the array to collectively capture a broad spectrum of urinary VOC signatures. The unit heats the tab to 120 °C, releasing the adsorbed volatiles, and sensor responses to the desorbed vapours were recorded over 180 s, digitised, and stored. Individual sensor response was quantified as %ΔR/R, where R is the baseline resistance in clean air and ΔR is the change in resistance upon exposure to the headspace volatiles. The resulting multivariate response constitutes a chemical “fingerprint” of the VOC mixture, which can be analysed using chemometric techniques or neural network-based pattern recognition to discriminate between normal and abnormal patient groups and to estimate the likelihood of specific disease states for which the instrument has been trained [16].

For each sample, the segment of the sensor response corresponding to volatile desorption was extracted and averaged to generate five representative data points spanning the response profile. Features derived from the raw sensor data were normalised using z-score normalisation and compiled into pattern databases, which served as the basis for subsequent analyses. To visualise global structure within the dataset, principal components analysis (PCA) [17] was applied, providing an unbiased means of identifying clustering or separation between patient and control populations.

Normality of individual sensor responses was assessed using the Shapiro–Wilk test for each group. As all features showed significant deviation from normality, sensor responses were compared between HCC and cirrhosis groups using the non-parametric Mann–Whitney U test, applied independently for each of the eight sensors, with Cliff’s δ reported as the effect size measure.

### 2.6. Statistical Analysis

Demographic and clinical variables were analysed using appropriate statistical tests. Continuous variables were expressed as mean ± standard deviation. Categorical variables were presented as frequencies and percentages. Group comparisons employed Mann–Whitney U tests for continuous variables and chi-square tests for categorical variables. OriginPro (Version 2024). OriginLab Corporation, Northampton, MA, USA was used for PCA, while ROC analysis and precision–recall were calculated using MedCalc^®^ Statistical Software version 23.5.9 (MedCalc Software Ltd., Ostend, Belgium; https://www.medcalc.org; accessed on 23 June 2026). Proprietary programmes were written for neural networks utilised.

Principal components analysis (PCA) was employed for unsupervised dimensionality reduction and visualisation of VOC sensor data, providing an unbiased means of observing clustering between HCC and cirrhosis populations. PCA was conducted on normalised sensor response data, with 95% confidence ellipses constructed to delineate cluster boundaries.

Following TRIPOD-AI guidelines, four supervised learning models were implemented within a unified evaluation framework: a class-weighted radial basis function network (RBF), penalised one vs. rest logistic regression (LOGISTIC), linear one vs. rest support vector machine with Platt calibration (SVM), and a weighted Random Forest ensemble (RANDOMFOREST) (see Supplementary Information) [18]. All models support stratified k-fold cross validation, probability calibration, and polymorphic cloning. Class imbalance is addressed consistently across models using inverse frequency weighting. Calibration metrics (slope, intercept, Brier score) are computed using binned logit regression, and all models integrate seamlessly with the shared evaluation pipeline (see Supplementary Information for detailed descriptions). The RBF network was chosen based on the overall performance for supervised classification (see Tables S2 and S3).

All analyses used pre-processed sensor measurements paired with categorical class labels. Each sample consisted of a fixed-length feature vector extracted from the sensor array. No missing data was present. All samples were included in the analysis. The prediction target was the categorical class label associated with each sample. Ground-truth labels were obtained directly from the curated dataset and treated as the reference standard. No manual feature selection was performed; all features were used for model training. Predictors were not transformed beyond standard numerical representation.

All models were trained using stratified k-fold cross-validation (k = 5). Stratification ensured that each fold preserved class proportions. For each fold, models were trained on k–1 folds and evaluated on the held-out fold. After cross-validation, each model was retrained on the full dataset to produce a final deployable model. In practice, the neural network was trained on samples drawn from replicate tab 1 per patient. The trained network was subsequently validated against the complete second replicate tab for all participants—an entirely independent external validation set not used at any stage of training or cross-validation. This provided information about whether the trained neural network was robust against sample variation, albeit from the same patient population. Network architecture and hyperparameters were fixed prior to external validation. Classification performance was evaluated using sensitivity, specificity, PPV, NPV, positive and negative likelihood ratios, and AUROC, all with 95% confidence intervals. ROC analysis was performed using the DeLong method; the precision–recall curve was additionally generated given the class imbalance inherent in surveillance populations.

## 3. Results

### 3.1. Study Population Characteristics

A total of 256 participants were recruited across the four study centres, comprising 72 (28.1%) HCC patients and 184 (71.9%) cirrhosis controls. The geographical distribution demonstrated: UHCW contributed 26 (36.1%) HCC and 91 (49.5%) cirrhosis patients; Royal Stoke contributed 24 (33.3%) HCC and 24 (13.0%) cirrhosis patients; New Cross Hospital contributed 8 (11.1%) HCC and 44 (23.9%) cirrhosis patients; and RHH contributed 14 (19.4%) HCC and 25 (13.6%) cirrhosis patients.

Demographic characteristics revealed significant sex distribution differences between cohorts. Males comprised 80.6% (58/72) of the HCC cohort compared to 59.8% (110/184) of the cirrhosis cohort (*p* = 0.002, chi-square test). Male sex was associated with significantly elevated HCC risk (relative risk 2.17, 95% CI: 1.29–3.66), whereas female sex demonstrated protective association (relative risk 0.78, 95% CI: 0.68–0.90) (Table 1).

BMI distribution was broadly comparable between groups, with no statistically significant differences. Among HCC patients with available BMI data (*n* = 57), 19.3% were normal weight, 36.8% overweight, 31.6% obese, and 12.3% morbidly obese. Among cirrhosis patients (*n* = 181), the distribution was: 0.6% underweight, 16.0% normal weight, 32.0% overweight, 27.6% obese, and 23.8% morbidly obese.

Smoking prevalence demonstrated no significant difference between cohorts: 27.8% (20/72) of HCC patients versus 26.1% (48/184) of cirrhosis patients were current or former smokers (*p* = 0.783). Similarly, alcohol consumption history showed comparable rates: 30.6% (22/72) HCC patients versus 31.0% (57/184) cirrhosis patients (*p* = 0.984).

### 3.2. Aetiological Distribution

Liver disease aetiology varied substantially between cohorts. In the HCC cohort, cryptogenic cirrhosis predominated (50.0%, 36/72), followed by alcohol-related liver disease (12.5%, 9/72) and MASLD (15.3%, 11/72). Viral hepatitis was uncommon, with hepatitis B and C each representing 1.4% (1/72). Notably, 8.3% (6/72) of HCC cases were incidental findings without prior known cirrhosis.

Conversely, in the cirrhosis cohort without HCC, alcohol-related liver disease constituted the predominant aetiology (48.4%, 89/180), followed by MASLD (31.7%, 57/180). Cryptogenic cirrhosis accounted for only 8.2% (15/180) of the cirrhosis cohort, representing a striking contrast to the HCC population. Autoimmune hepatitis (4.3%, 8/180), primary biliary cholangitis (2.7%, 5/180), and other less common aetiologies comprised the remainder.

### 3.3. HCC Treatment Status

Among the HCC cohort, 50.0% (36/72) were untreated or awaiting treatment at the time of sample collection. Treatment modalities among treated patients included: trans-arterial chemoembolization (TACE, 26.4%, 19/72), immunotherapy (6.9%, 5/72), radiofrequency ablation (5.6%, 4/72), hepatectomy (4.2%, 3/72), and systemic chemotherapy (1.4%, 1/72). Six patients (8.3%) were being considered for liver transplantation.

### 3.4. Biochemical Parameters

Biochemical comparison between HCC and cirrhosis cohorts revealed several statistically significant differences. Serum AFP demonstrated marked elevation in HCC patients (1390.45 ± 7365.39 KU/L) compared to cirrhosis patients (87.51 ± 799.83 KU/L, *p* < 0.001), consistent with its established association with HCC. ALT levels were significantly higher in HCC (47.09 ± 30.37 U/L) versus cirrhosis (36.58 ± 26.03 U/L, *p* = 0.003). Similarly, ALP demonstrated elevation in HCC (182.89 ± 194.09 U/L) compared to cirrhosis (132.14 ± 66.38 U/L, *p* = 0.008).

Conversely, serum albumin was significantly lower in HCC patients (34.45 ± 7.00 g/L) compared to cirrhosis patients (36.70 ± 8.64 g/L, *p* = 0.024), potentially reflecting more advanced liver dysfunction in the HCC cohort. Parameters including INR (HCC: 1.19 ± 0.21 vs. cirrhosis: 1.22 ± 0.49, *p* = 0.275), prothrombin time (HCC: 13.37 ± 3.32 s vs. cirrhosis: 15.16 ± 6.66 s, *p* = 0.504), and GGT (HCC: 277.19 ± 281.50 U/L vs. cirrhosis: 194.18 ± 190.36 U/L, *p* = 0.494) showed no significant differences between groups (Table 2).

### 3.5. Urinary VOC Profiling Results

**Figure 2:**
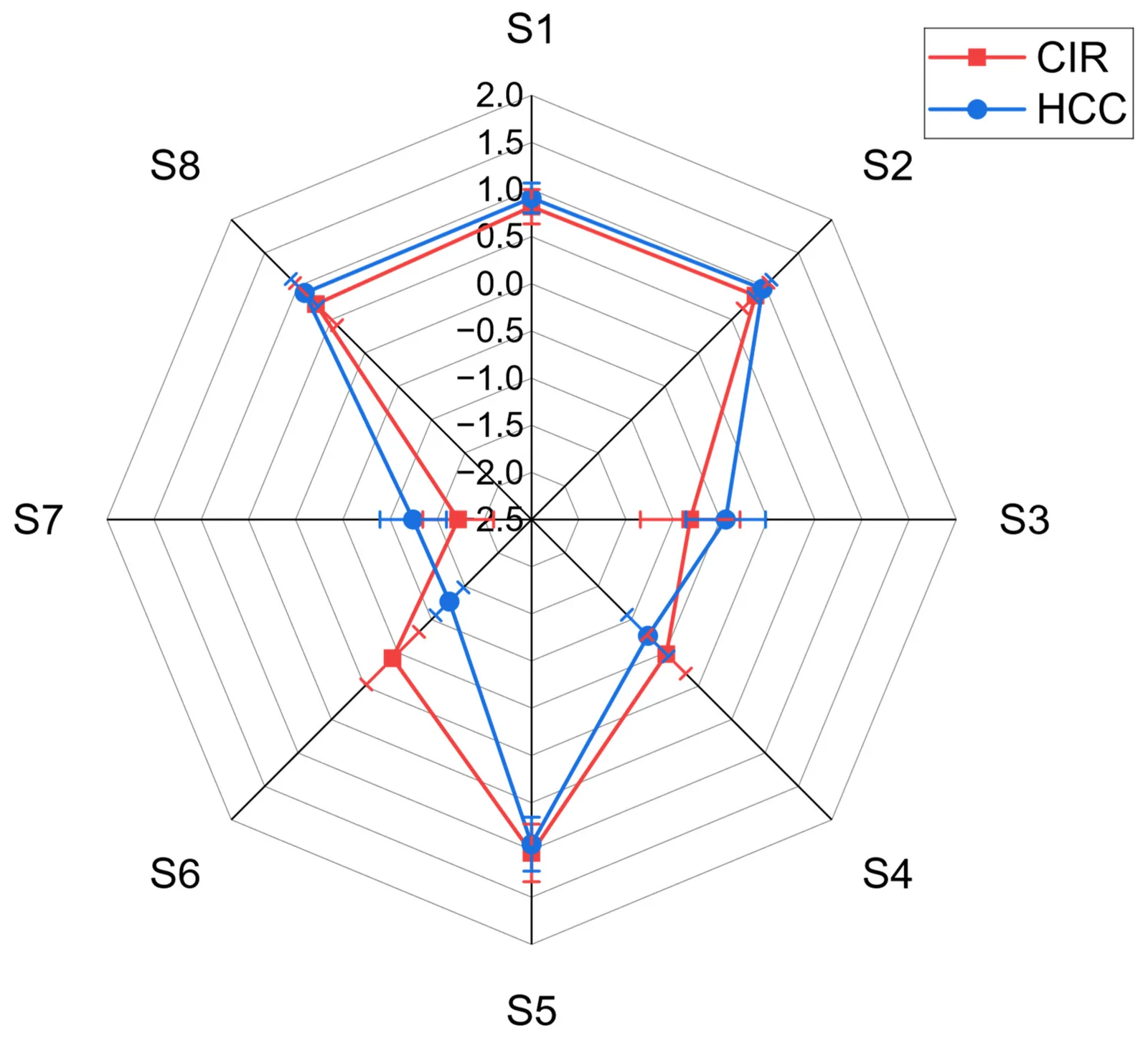
Radar plot of normalised responses from an array of eight conducting-polymer metal oxide gas sensors to urine headspace vapours from hepatocellular carcinoma (HCC) and cirrhosis (CIR) participants, averaged across all participant samples. Each axis represents one sensor (S1–S8). The larger the sensor response, the further the trace extends from the centre. Divergence between the HCC (red) and CIR (blue) traces indicates differential sensor reactivity to the distinct volatile organic compound (VOC) profiles of each group.

VOC analysis was successfully performed on 190 participants (58 HCC, 132 cirrhosis). Individual sensor responses demonstrated significant discrimination between HCC and cirrhosis cohorts. Figure 2 shows a radar plot of normalised sensor responses and standard deviations derived from an array of eight metal oxide gas sensors. Normality of each sensor feature was assessed using the Shapiro–Wilk test for CIR and HCC groups. All features showed extreme deviation from normality, so a non-parametric Mann–Whitney U test was applied to test the effect of different sensors in discriminating between the groups (Table 3). The strongest potential discriminators were S3, S4, S6, S7, S8, moderate discriminators were S1, S2, and the weakest was S5. As these gas sensors respond to a broad spectrum of volatile chemicals, with each sensor responding to a slightly different range of compounds, it is not possible to deduce what is chemically different between the headspace of HCC and cirrhosis samples.

### 3.6. Principal Components Analysis

**Figure 3:**
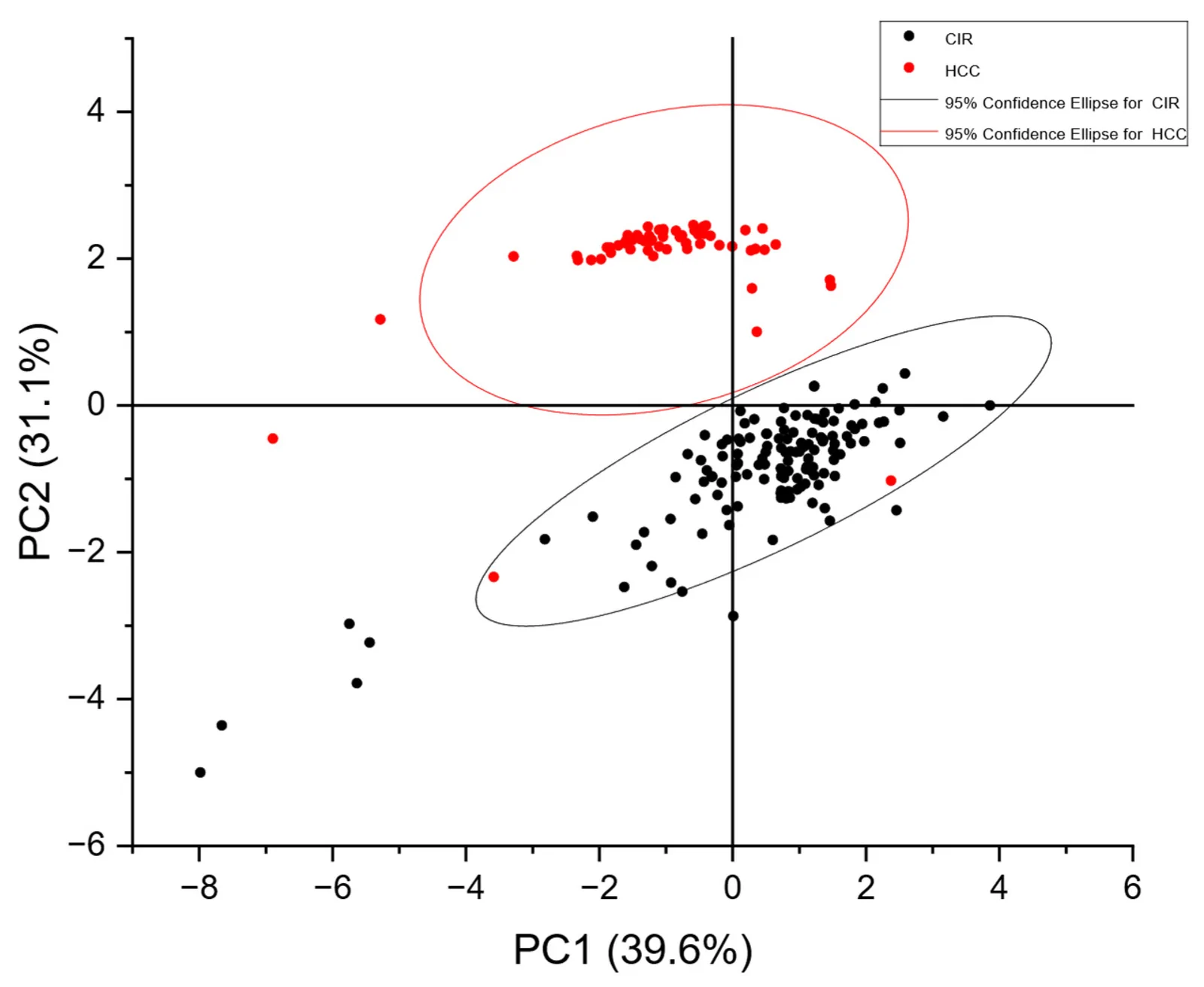
Principal components analysis (2D) showing clear separation between HCC and cirrhosis populations. The first two principal components account for 70.7% of total variance. Ellipses represent 95% confidence intervals. Two HCC samples (HCC019, HCC013) fall within the cirrhosis cluster region.

**Figure 4:**
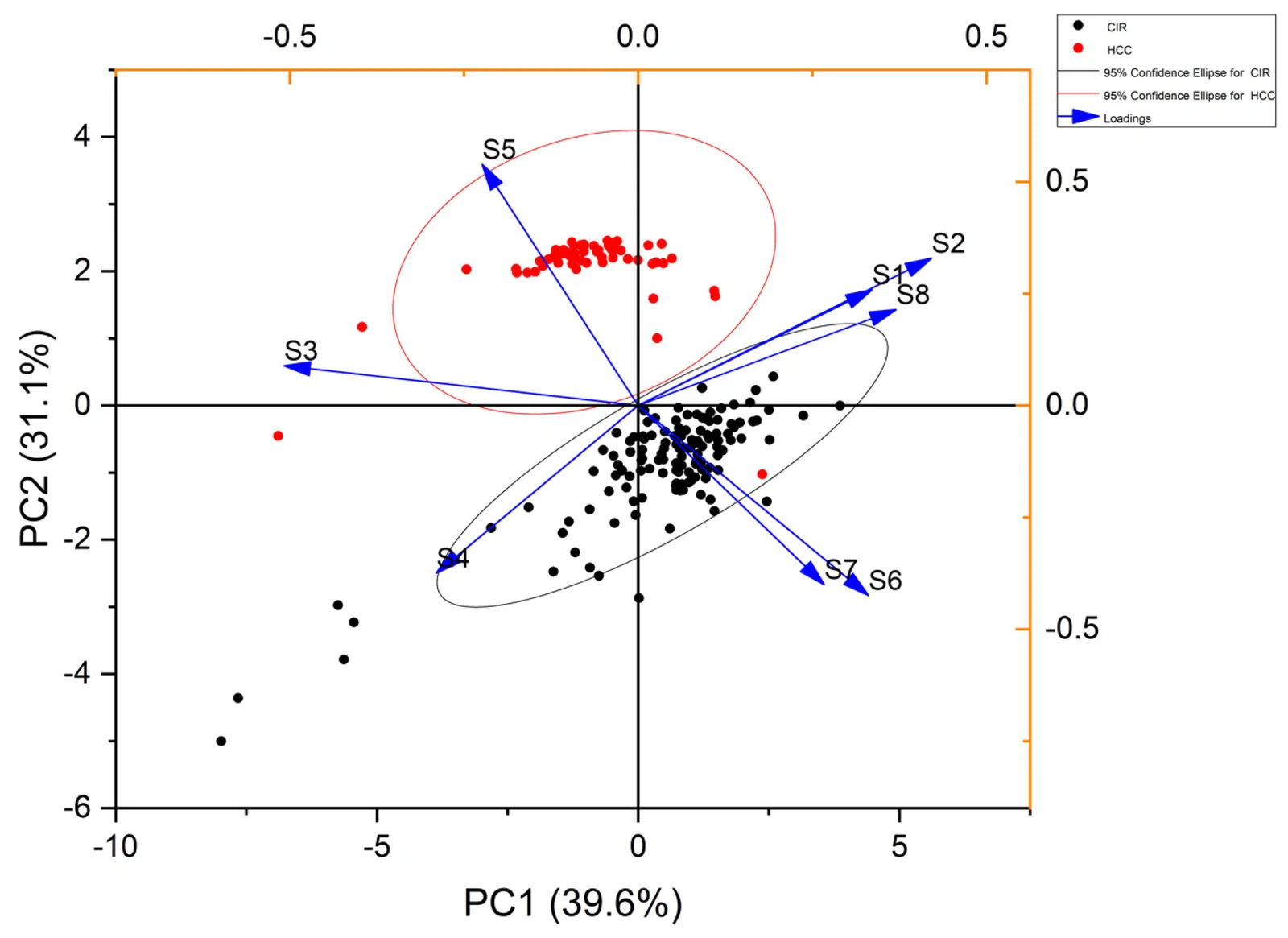
PCA loadings plot superimposed on the two-dimensional PCA scatter plot, illustrating the contribution of each sensor (S1-S8) to class discrimination. Arrow length and direction indicate the magnitude and orientation of each sensor’s contribution to the principal component axes. Sensors S3, S5, S6, and S7 demonstrate the greatest contribution to HCC-cirrhosis discrimination along PC2, reflecting their strongest covariance with the class-separating component.

**Figure 5:**
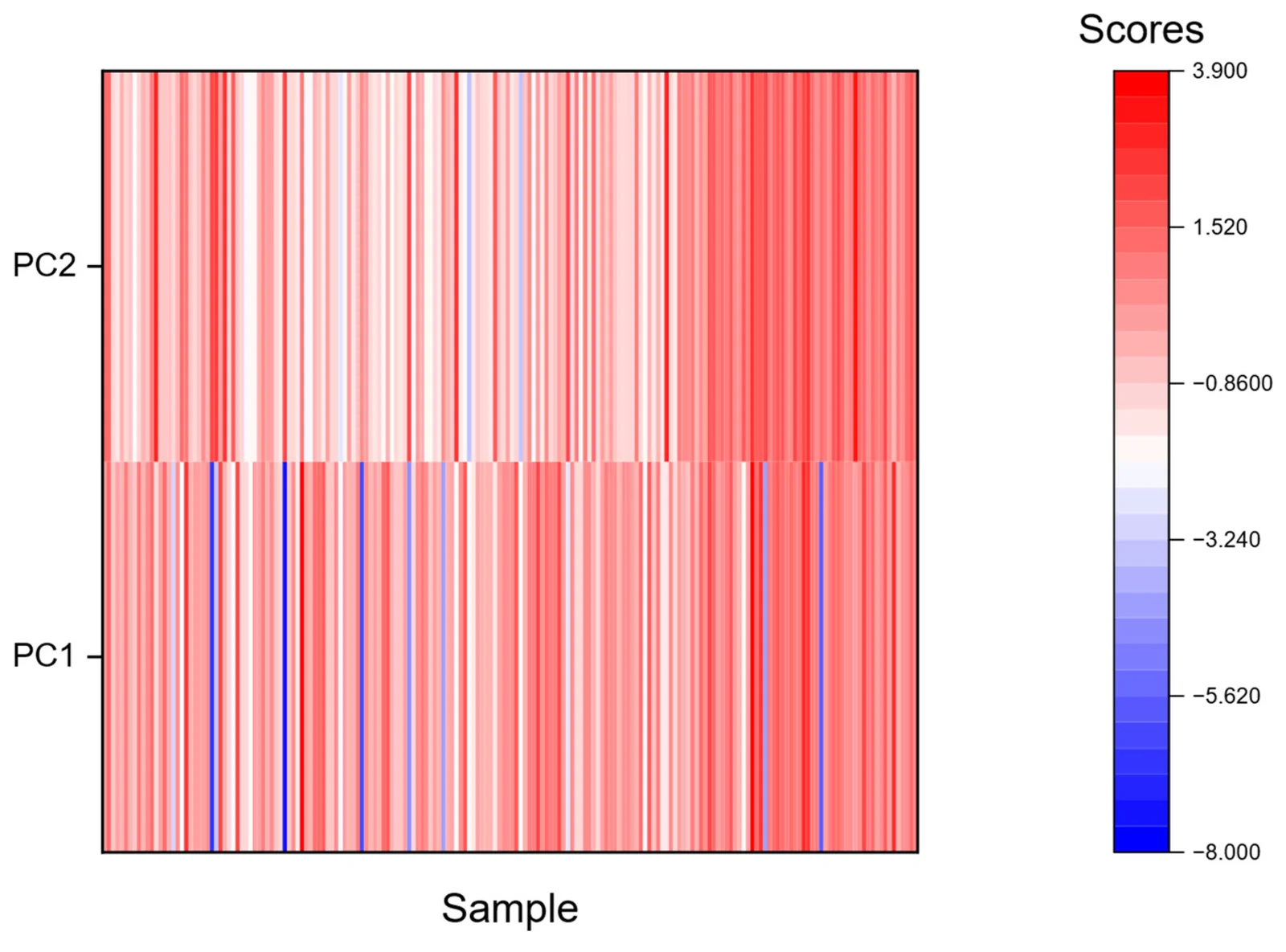
Heatmap of principal component scores for each participant sample (rows), showing the relative contribution of PC1 and PC2 (columns) to class discrimination between HCC and cirrhosis populations. Warmer colours indicate higher positive scores; cooler colours indicate negative scores. PC2 demonstrates the greatest contribution to group separation.

PCA of the VOC dataset revealed distinct clustering between HCC and cirrhosis populations. The first two principal components accounted for 70.7% of the total variance in the dataset, with clear separation between groups evident within 95% confidence ellipses. Between-group separation was confirmed by PERMANOVA (Pseudo-F: 228.4, *p*-value: 0.001, R^2^: 0.54). The between-group distances (CIR vs. HCC) given by Pseudo-F are vastly larger than the within-group distances. Two HCC samples (HCC019, HCC013) were identified within the cirrhosis cluster region (Figure 3 and Figure 4). A heatmap of the principal component scores for each participant sample (Figure 5) indicated that PC2 contributed greatly to the discrimination between classes. Loadings analysis identified sensors S3, S5, S6, and S7 as primary contributors to class discrimination along PC2, even though S5 was the weakest contributor from Table 3. This is common for PCA as it captures patterns. A single sensor can display a small mean difference between classes, but show strong covariance with other sensors, and therefore contribute to a discriminating PC.

**Figure 6:**
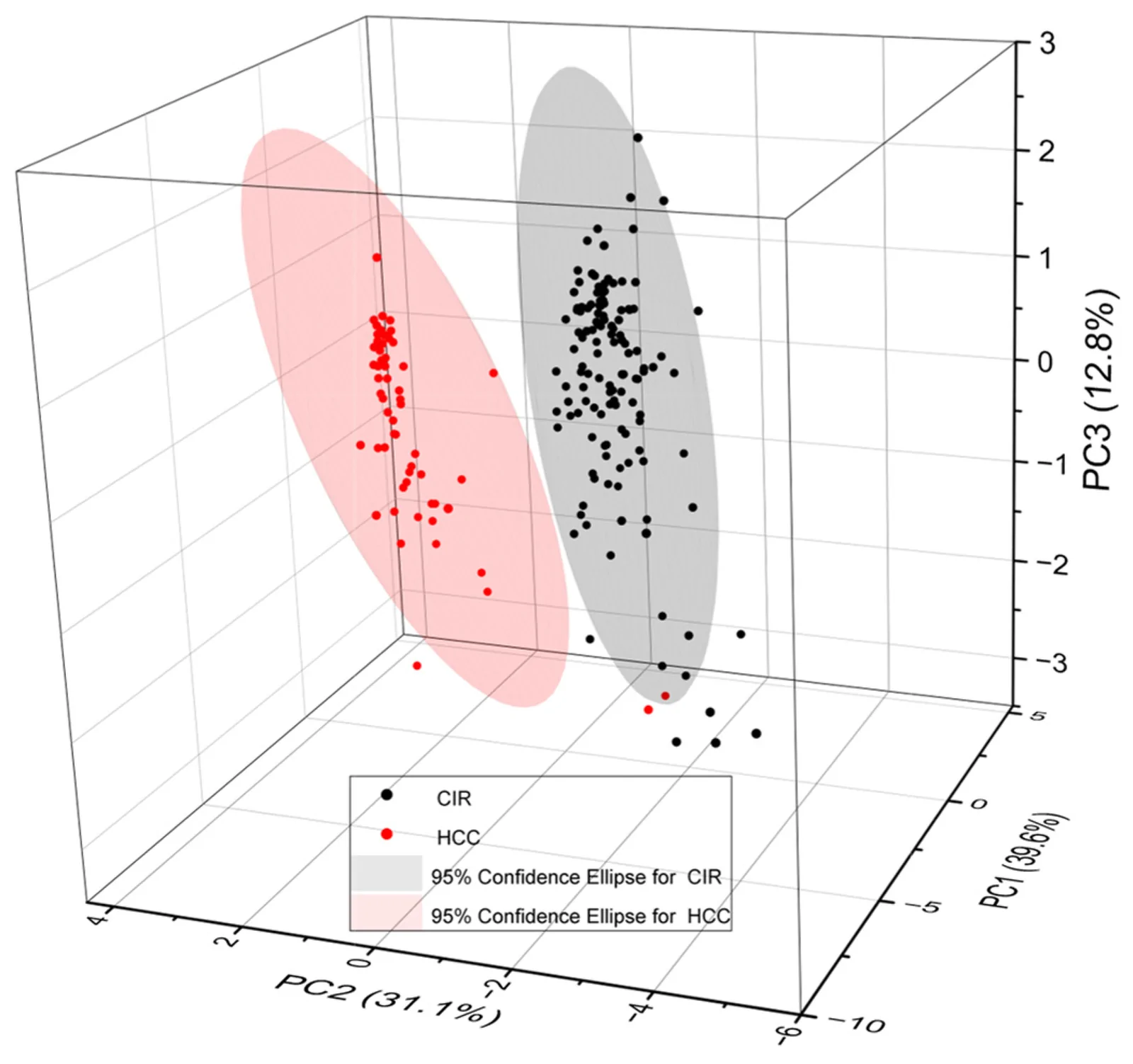
Three-dimensional PCA plot with 95% confidence ellipses. The third principal component accounts for an additional 12.8% of variance (cumulative 83.5%). The plot has been rotated to optimise visualisation of cluster separation.

Three-dimensional PCA (Figure 6) incorporating the third principal component (accounting for an additional 12.8% of variance, cumulative 83.5%) further enhanced visualisation of population separation.

### 3.7. Neural Network Classification Performance

Across the four evaluated architectures (Tables S2 and S3), probability calibration and error-pattern analysis consistently favoured the radial basis function network. The RBF model showed near-perfect calibration (slope 0.991, intercept 0.027) and highly accurate probability estimates, reflected in its low Brier score of 0.0131, whereas alternative models demonstrated characteristic biases. Logistic regression showed mild overconfidence, SVM underconfidence and the least accurate probabilities, and Random Forest probability compression despite the lowest Brier score. McNemar comparisons further indicated that RBF made statistically distinct decisions from Logistic, SVM, and Random Forest (*p* = 0.004–0.002), confirming that it captured different diagnostic boundaries rather than reproducing the same error structure. Together with its high external-validation performance, these findings support the choice of the RBF network for this application.

**Figure 7:**
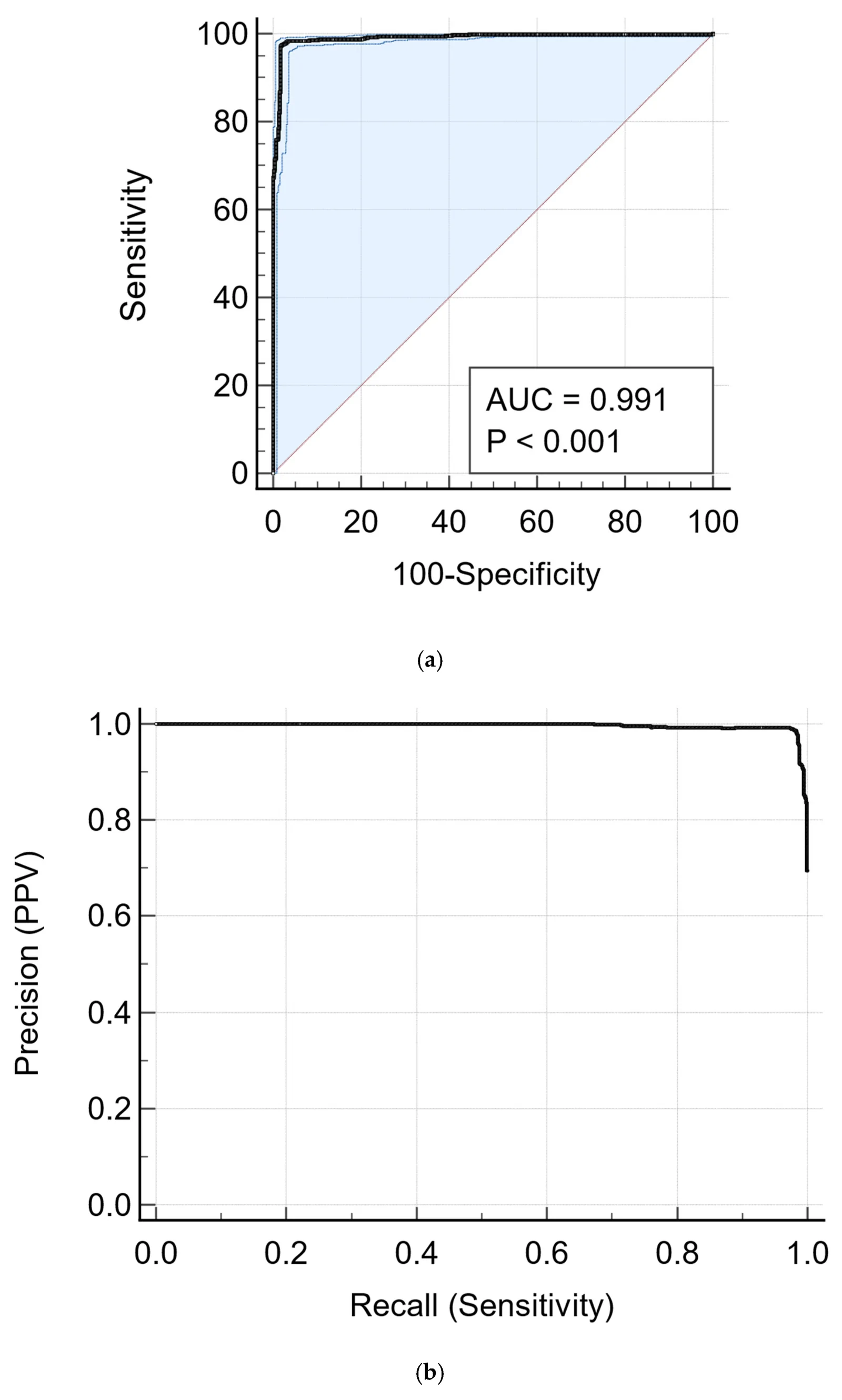
(**a**) Receiver operating characteristic (ROC) curve (black solid line) with 95% confidence interval (light blue shaded area) and diagonal reference line representing chance-level discrimination (red dashed line). AUROC = 0.991 (95% CI: 0.986–0.995), indicating near-perfect discrimination between HCC and cirrhosis populations. (**b**) Precision–recall curve (black solid line) for HCC vs. cirrhosis classification (AUROC = 0.996, 95% CI: 0.991–0.998). This demonstrates robust classifier performance despite class imbalance in the dataset.

The RBF neural network was trained using a 20/80 stratified data split, with 20% of samples (from replicate tab 1) used for training and 80% reserved for internal validation. A five-fold cross-validation (CV) strategy was applied within the training set to optimise network parameters and minimise overfitting. The trained network was then validated on an entirely independent dataset comprising the second replicate tab for all participants—data not seen at any stage of training or cross-validation. The confusion matrix from repeated iterations of training and testing typically gave 57 true positive HCC identifications, 128 true negative cirrhosis identifications, one false positive classification, and four false negative classifications (confusion value: 0.03); the full confusion matrix is reported in Supplementary Table S1. These classifier outputs over 5 K-folds were evaluated by ROC (Figure 7a) yielding diagnostic accuracy metrics summarised in Table 4.

The area under the curve (AUROC) was 0.991 (95% CI: 0.986–0.995, *p* < 0.0001), indicating strong discrimination between HCC and cirrhosis. These results compare favourably with published performance benchmarks for conventional surveillance modalities. Precision–recall curve analysis, particularly relevant given class imbalance in surveillance populations, demonstrated an area under the curve of 0.99 (Figure 7b).

The Youden index measures how well a test separates two classes, a random classifier has a J = 0, and for a perfect classifier J = 1. In this case J = 0.95 (CI: 0.94 to 0.97).

Table 4 also shows estimated specificity at fixed sensitivity and vice versa for different cut-off points on the ROC, and this indicates that VOC measurements should achieve greater than 80% specificity and sensitivity in practical measurement scenarios.

## 4. Discussion

This multicentre prospective study demonstrates that urinary VOC profiling achieves high diagnostic accuracy in distinguishing HCC from cirrhosis. As seen from Table 4 (derived from the receiver operating curve), fixing the sensitivity at 80% would potentially achieve 98.6% specificity, while fixing specificity at 80% would potentially achieve 98.8% sensitivity—a performance substantially exceeding that of current surveillance modalities. The AUROC of 0.99 represents good discrimination, whilst the precision–recall curve confirms robust performance even with class imbalance typical of surveillance populations. These findings suggest that VOC-based diagnostics holds potential to address critical unmet needs in HCC early detection.

For surveillance of populations, the optimal operating point lies toward the high-sensitivity region of the ROC curve, where the model prioritises detection of early HCC even at the expense of additional false positives. This reflects the clinical reality that the consequences of a missed early-stage disease where loss of curative treatment eligibility and markedly reduced survival would be substantially more severe than the burden of caring for cirrhotic patients. The calibration of the RBF network (slope 0.991, intercept 0.027) ensures that probability estimates remain trustworthy even in this high-sensitivity region, supporting its use as a risk-stratification tool in routine surveillance. However, this also highlights the need for calibration when transferring to other measurement contexts, and there is the danger of overfitting data based on limited data sets. The current work reaches TRIPOD-AI Type 2 standards (development + internal validation).

The present findings should also be contextualised against emerging biomarker strategies under evaluation for HCC surveillance. The GALAD score, which incorporates gender, age, AFP-L3, AFP, and des-gamma-carboxyprothrombin (DCP), has been proposed as a composite serum biomarker for the detection of hepatocellular carcinoma. In this study, GALAD demonstrated an AUC of 0.91 and, at a threshold of -0.63, showed a sensitivity of 68% and a specificity of 95% [20]. Circulating tumour DNA and cell-free DNA approaches (liquid biopsy) offer high specificity but remain constrained by low sensitivity at early tumour stages and high cost, limiting applicability in routine surveillance programmes [21]. In contrast, the urinary VOC platform evaluated in the present study offers important practical advantages: urine collection is entirely non-invasive, operator-independent, and potentially amenable to point-of-care deployment in resource-limited settings where access to AFP assays, imaging, and liquid biopsy infrastructure is restricted. Critically, unlike GALAD and liquid biopsy approaches which require venepuncture and laboratory processing, the VOC platform produces results within minutes of sample analysis.

The potential diagnostic advantage of VOC profiling over existing surveillance methods, based on comparison with published literature benchmarks, is clinically significant. Current first-line surveillance with ultrasound achieves modest sensitivity of 45% (95% CI: 30–62%) for early-stage HCC detection [7], whereas a serum AFP threshold of 20–100 ng/mL demonstrates 61% sensitivity (95% CI: 60–62%) [8]. Even when combined, ultrasound with AFP achieves only 63% sensitivity (95% CI: 48–75%) for early HCC detection [7]. Further there is the added cost and patient inconvenience in having a two-stage screening modality.

In contrast, VOC profiling in the present prospective multi-centre study achieved 98% sensitivity (95% CI: 90.76% to 99.96%), representing a substantial improvement over current modalities and potentially translating to detection of many early-stage HCC cases currently missed by conventional surveillance. The concurrent specificity range reported in Table 4 minimises false-positive results, thereby reducing unnecessary anxiety, additional investigations, and healthcare expenditure associated with surveillance false alarms.

Principal component analysis is a dimensionality-reduction method that transforms correlated variables into a smaller set of orthogonal components that capture the maximum variance in the data. It provides an unbiased means of visualising whether distinct population clusters exist [17]. The clear HCC–cirrhosis separation within 95% confidence ellipses, with 70.7% of variance explained by the first two components, confirms that the sensor array detects a genuinely distinct VOC signature between the two groups. Sensors S3, S4, S6, and S7 were the primary discriminators, but all sensors in the array contributed, and this will inform future compound identification work. Two samples (HCC013, HCC019) fell within the cirrhosis cluster, likely because these patients had concurrent cirrhosis and treated HCC, shifting their VOC profile away from active cancer. This observation aligns with prior evidence that VOC levels differ between treated and partially treated HCC patients [10], suggesting that VOC profiling may be most sensitive for active untreated disease and raising the possibility that VOC profiles could serve as markers of treatment response—an avenue warranting prospective evaluation. The Pseudo-F in PERMANOVA is a test statistic that measures how much between-group multivariate variation exceeds within-group variation, and the result confirmed clear robust separation between CIR and HCC, large effect size, highly significant group differences, and low overlap in multivariate space.

The biological basis for VOC-based HCC detection likely reflects fundamental metabolic alterations accompanying hepatocarcinogenesis. Malignant transformation profoundly alters cellular metabolism, with the Warburg effect, enhanced lipogenesis, altered amino acid metabolism, and dysregulated cytochrome P450 activity potentially contributing to distinctive VOC signatures [9]. The sensors demonstrating greatest discriminatory capacity presumably detect specific volatile metabolites enriched or depleted in HCC compared to cirrhosis. Our pilot study identified several candidate compounds including 2-butanone, 2-hexanone, and benzene derivatives [12], though definitive compound identification requires complementary gas chromatography–mass spectrometry analysis currently underway.

The demographic and clinical characteristics of our cohort warrant consideration. The male predominance in the HCC cohort (80.6% vs. 59.8% in cirrhosis, relative risk 2.17) aligns with established epidemiological patterns, reflecting higher HCC incidence among males across diverse populations [1]. The striking aetiological differences between cohorts—with cryptogenic cirrhosis predominating in HCC (50.0%) but accounting for only 8.2% of cirrhosis controls—may reflect ascertainment bias, as patients with advanced or decompensated cirrhosis of known aetiology may undergo more intensive surveillance. Alternatively, this pattern could suggest biological differences in HCC risk between cryptogenic and alcohol-related or MASLD-driven cirrhosis, though this requires prospective validation.

Several study limitations merit acknowledgment. First, a healthy control cohort was not included, as the study was deliberately designed to address the clinically relevant question of distinguishing HCC from established cirrhosis—the population in whom surveillance is indicated. Whilst the Bannaga et al. pilot study demonstrated VOC discrimination between HCC and non-HCC patients [12], the present study’s comparator was intentionally set as cirrhotic controls to reflect real-world surveillance conditions [6]. Whether VOC profiling can additionally distinguish cirrhosis from healthy liver warrants evaluation in future studies incorporating a three-arm design. Second, the HCC cohort included both treated and untreated patients—with 50% untreated or awaiting treatment, 26.4% having received TACE, and a subset receiving systemic therapy—and therapeutic interventions could potentially alter VOC results, as has been demonstrated for exhaled VOCs in treated versus partially treated HCC. Future studies restricted to untreated HCC samples compared against cirrhosis and healthy controls would help validate the full discriminatory scope of this approach. Third, the study captures a single time-point measurement per participant; longitudinal assessment of VOC profiles across disease progression and treatment would be required to establish the dynamic behaviour of these signatures and their potential utility for treatment response monitoring. Fourth, incomplete demographic data capture—particularly age in the cirrhosis cohort and BMI across both groups—reflects the pragmatic constraints of conducting a multicentre study with limited dedicated research infrastructure. Importantly, the classification model was built exclusively on VOC sensor response data and did not incorporate demographic variables, so missing demographic data does not affect the diagnostic model itself; however, it does limit the extent of covariate-adjusted analyses. Validation across geographically and aetiologically diverse cohorts—particularly those where viral hepatitis predominates as the cause of cirrhosis and HCC—is needed before the findings can be generalised more widely.

Whilst cost-effectiveness analysis was not performed, the eight-sensor platform offers potential advantages over imaging modalities, and formal health economic evaluation comparing VOC screening against standard surveillance protocols is required to inform policy decisions.

Despite these limitations, several strengths support the validity and clinical relevance of our findings. The multicentre design enhances generalisability compared to single-centre studies. The sample size calculated using rigorous statistical methodology and representing substantial expansion over previous VOC studies provides adequate statistical power for robust conclusions. The use of duplicate measurements and independent test sets in neural network validation minimises overfitting concerns. The exceptional diagnostic performance metrics, particularly the combination of high sensitivity and high specificity, suggest genuine biological signal rather than statistical artefact.

The clinical implementation pathway for VOC-based HCC surveillance requires systematic validation. Immediate priorities include: (1) prospective validation in independent cohorts with longitudinal follow-up to assess surveillance interval performance; (2) three-way classification studies incorporating healthy controls; (3) assessment of VOC profile evolution during disease progression and treatment; (4) compound identification through comprehensive gas chromatography–mass spectrometry; (5) development of point-of-care devices for widespread deployment; (6) cost-effectiveness analysis comparing VOC surveillance against standard protocols; and (7) regulatory pathway evaluation for clinical diagnostic approval.

The potential advantages of VOC-based surveillance extend beyond diagnostic accuracy. The non-invasive nature of urine sampling offers superior patient acceptability compared to venepuncture, potentially improving surveillance adherence rates—a recognised challenge in current programmes. The absence of operator-dependency eliminates the inter-observer variability inherent in ultrasound imaging. The rapid analysis timeframe (minutes rather than days for conventional tests) facilitates same-visit results, potentially accelerating diagnostic pathways. The relatively low complexity of the analytical platform compared to advanced imaging modalities suggests feasibility for point-of-care deployment, particularly valuable in resource-limited settings where HCC burden is highest but access to ultrasound and MRI is restricted.

Integration with existing surveillance algorithms warrants consideration. VOC testing could serve as: (1) a primary screening tool replacing ultrasound in selected populations; (2) a triage tool to identify patients requiring urgent imaging; (3) an adjunct to ultrasound for improved sensitivity; or (4) a monitoring tool for treatment response assessment. Given the 6-month surveillance interval recommended by international guidelines [6], the stability of VOC signatures over this timeframe and optimal testing frequency require empirical evaluation.

In conclusion, urinary VOC profiling demonstrates good diagnostic accuracy for distinguishing HCC from cirrhosis in this multicentre study, substantially outperforming current surveillance modalities. The exceptional sensitivity and specificity, combined with non-invasive sampling, rapid analysis, and potential for point-of-care deployment, position VOC-based diagnostics as a highly promising approach for HCC surveillance in high-risk cirrhotic populations. Further validation in large prospective cohorts and clinical implementation studies is warranted to establish this technology as a transformative advance in HCC early detection.

## Figures and Tables

**Table 1:** Baseline demographic and clinical characteristics.

Characteristic	HCC (*n* = 72)	Cirrhosis (*n* = 184)	*p*-Value
**Age (years)**			
Mean ± SD	71.4 ± 9.0	61.6 ± 10.1	<0.001 ^b^
Median (IQR)	73 (65–79)	62 (57–69)	<0.001 ^b^
Range	46–87	35–80	
*Missing data*	*7/57 (12.3%) ^a^*	*112/184 (60.9%) ^a^*	
**Sex, *n* (%)**			
Male	58 (80.6)	110 (59.8)	0.002 ^c^
Female	14 (19.4)	74 (40.2)	
*Missing data*	*0/72 (0%)*	*0/184 (0%)*	
**BMI category, *n* (%),** *HCC n = 57; CIR n = 181*	**HCC (*n* = 57)**	**CIR (*n* = 181)**	***p*-value**
Underweight (<18.5)	0 (0)	1 (0.6)	
Normal weight (18.5–24.9)	11 (19.3)	29 (16.0)	
Overweight (25.0–29.9)	21 (36.8)	58 (32.0)	
Obese (30.0–39.9)	18 (31.6)	50 (27.6)	
Morbidly obese (≥40.0)	7 (12.3)	43 (23.8)	0.145 ^c^
*Missing data*	*15/72 (20.8%) ^a^*	*3/184 (1.6%) ^a^*	
**Smoking history, *n* (%)**			
Current or former smoker	20 (27.8)	48 (26.1)	0.783 ^c^
Never/unknown	52 (72.2)	136 (73.9)	
*Missing data*	*0/72 (0%)*	*0/184 (0%)*	
**Alcohol consumption history, *n* (%)**			
Yes	22 (30.6)	57 (31.0)	0.984 ^c^
No	50 (69.4)	127 (69.0)	
*Missing data*	*0/72 (0%)*	*0/184 (0%)*	
**Liver disease aetiology, *n* (%),** HCC *n* = 72; CIR *n* = 180	**HCC (*n* = 72)**	**CIR (*n* = 180)**	***p*-value**
Cryptogenic cirrhosis	36 (50.0)	15 (8.3)	<0.001 ^c^
Alcohol-related liver disease	9 (12.5)	89 (49.4)	<0.001 ^c^
MASLD	11 (15.3)	57 (31.7)	0.008 ^c^
Autoimmune hepatitis	1 (1.4)	8 (4.4)	0.453 ^c^
Hepatitis B	1 (1.4)	2 (1.1)	1.000 ^c^
Hepatitis C	1 (1.4)	1 (0.6)	0.491 ^c^
Primary biliary cholangitis	0 (0)	5 (2.8)	0.326 ^c^
Incidental finding (no known CLD)	6 (8.3)	1 (0.6)	0.003 ^c^
Genetic (e.g., haemochromatosis)	0 (0)	2 (1.1)	1.000 ^c^
Missing data	7/72 (9.7%) ^a^	0/180 (0%)	
**Recruitment centre, *n* (%)**			
UHCW	26 (36.1)	91 (49.5)	
Royal Stoke University Hospital	24 (33.3)	24 (13.0)	
New Cross Hospital	8 (11.1)	44 (23.9)	
Russell’s Hall Hospital	14 (19.4)	25 (13.6)	
Missing data	0/72 (0%)	0/184 (0%)	

**Notes:** ^a^ Missing data from source dataset. ^b^ Mann–Whitney U test (two-sided). ^c^ Chi-square test; Fisher’s exact used for cells with expected count <5. MASLD = metabolic dysfunction-associated steatotic liver disease; NAFLD = non-alcoholic fatty liver disease. UHCW = University Hospitals Coventry and Warwickshire.

**Table 2:** Biochemical parameters by cohort.

Biochemical Parameter	HCC (*n* = 72) Mean ± SD	Cirrhosis (*n* = 184) Mean ± SD	*p*-Value
AFP (KU/L) ^a^	1390.45 ± 7365.39	87.51 ± 799.83	<0.001 ^b^
ALT (U/L)	47.09 ± 30.37	36.58 ± 26.03	0.003 ^b^
ALP (U/L)	182.89 ± 194.09	132.14 ± 66.38	0.008 ^b^
Albumin (g/L)	34.45 ± 7.00	36.70 ± 8.64	0.024 ^b^
INR	1.19 ± 0.21	1.22 ± 0.49	0.275 ^b^
Prothrombin time (s)	13.37 ± 3.32	15.16 ± 6.66	0.504 ^b^
GGT (U/L)	277.19 ± 281.50	194.18 ± 190.36	0.494 ^b^

**Notes:** ^a^ AFP highly right-skewed (SD exceeds mean); median (IQR) preferred—raw data required. ^b^ Mann–Whitney U test (two-sided); *p* < 0.05 statistically significant. AFP = alpha-fetoprotein; ALT = alanine aminotransferase; ALP = alkaline phosphatase; GGT = gamma-glutamyl transferase; INR = international normalised ratio.

**Table 3:** Mann–Whitney U test of normalised gas sensor responses.

Sensor	Mann–Whitney U	*p*-Value	Cliff’s δ (Effect Size)	Effect Size
**1**	2639	2.99 × 10^−4^	−0.327	Small–Medium
**2**	2172	8.21 × 10^−7^	−0.446	Medium–Large
**3**	1650	1.56 × 10^−10^	−0.579	Large
**4**	1955	3.01 × 10^−8^	+0.502	Large
**5**	3778	0.682	+0.037	Negligible
**6**	304	2.23 × 10^−24^	+0.923	Huge
**7**	1100	1.90 × 10^−15^	−0.720	Very Large
**8**	1908	1.40 × 10^−8^	−0.514	Large

**Table 4:** Neural network classification and ROC performance metrics.

Area Under the ROC Curve (AUC)			
Area under the ROC curve (AUC)			0.991
Standard Error ^a^			0.00182
95% Confidence interval ^b^			0.986 to 0.995
95% Bootstrap CI ^c^			0.987 to 0.994
z statistic			270.243
Significance level P (Area = 0.5)			<0.0001
^a^ DeLong et al. [19]. ^b^ Binomial exact. ^c^ BC_a_ bootstrap confidence interval (1000 iterations; random number seed: 928).			
**Youden index**			
Youden index J			0.9555
95% Confidence interval ^a^			0.9357 to 0.9666
Associated criterion			>0.491332
95% Confidence interval ^a^			>0.482481 to >0.491332
Sensitivity			97.27
Specificity			98.28
^a^ BC_a_ bootstrap confidence interval (1000 iterations; random number seed: 928).			
Estimated specificity at fixed sensitivity			
Sensitivity	Specificity	95% CI ^a^	Criterion
80.00	98.62	97.41 to 99.31	>0.62673
90.00	98.28	97.07 to 99.14	>0.57956
95.00	98.28	97.07 to 99.14	>0.550314
97.50	97.93	96.03 to 99.14	>0.481833
99.00	79.14	72.41 to 93.84	>0.373287
Estimated sensitivity at fixed specificity			
Specificity	Sensitivity	95% CI ^a^	Criterion
80.00	98.79	97.95 to 99.28	>0.375648
90.00	98.64	97.80 to 99.17	>0.396476
95.00	98.41	97.50 to 98.94	>0.429223
97.50	97.65	0.00 to 0.00	>0.4729815
99.00	75.91	0.00 to 0.00	>0.6390386
^a^ BC_a_ bootstrap confidence interval (1000 iterations; random number seed: 928).			

## Data Availability

The data that support the findings of this study are available from the corresponding author upon reasonable request, subject to appropriate ethical approvals and data sharing agreements. Software code is proprietary, with dependencies on commercial software packages, and is not available.

## Supplementary Materials

The following supporting information can be downloaded at: https://www.mdpi.com/article/10.3390/s26185907/s1, Table S1: Confusion Matrix and Derived Performance Metrics—RBF Neural Network Classifier. Table S2: Calibration Results from 4 Neural Network Models tested. Table S3: Comparison of performance between different neural network architectures.

## Abbreviations

AASLD, American Association for the Study of Liver Disease; AFP, alpha-fetoprotein; AIH, autoimmune hepatitis; ALP, alkaline phosphatase; ALT, alanine aminotransferase; AUROC, area under the receiver operating characteristic curve; BCLC, Barcelona Clinic Liver Cancer; BMI, body mass index; CIR, cirrhosis; EASL, European Association for the Study of the Liver; FN, false negative; FP, false positive; GC-MS, gas chromatography-mass spectrometry; GC-TOF-MS, gas chromatography time-of-flight mass spectrometry; GGT, gamma-glutamyl transferase; HCC, hepatocellular carcinoma; INR, international normalised ratio; LR+, positive likelihood ratio; LR-, negative likelihood ratio; MASLD, metabolic dysfunction-associated steatotic liver disease; NAFLD, non-alcoholic fatty liver disease; NASH, non-alcoholic steatohepatitis; NCPH, non-cirrhotic portal hypertension; NICE, National Institute for Health and Care Excellence; NPV, negative predictive value; PBC, primary biliary cholangitis; PCA, principal component analysis; PPV, positive predictive value; RBF, radial basis function; RHH, Russells Hall Hospital; ROC, receiver operating characteristic; SPME, solid phase microextraction; TACE, transarterial chemoembolization; TN, true negative; TP, true positive; UHCW, University Hospitals Coventry and Warwickshire; VOC, volatile organic compound; WHO, World Health Organization.
