# Supplementary material for: Urinary Volatile Organic Compounds as Non-Invasive Biomarkers to Distinguish Hepatocellular Carcinoma from Cirrhosis: A Multicentre Diagnostic Accuracy Study
Source: Sensors (Basel). 2026 Sep 18;26(18):5907. doi: 10.3390/s26185907 (PMC13611087; doi:10.3390/s26185907)
Supplement: Supplementary file 1 [file sensors-26-05907-s001.zip › sensors-4483127-supplementary.pdf]

Article

Supplementary Table S1. Confusion Matrix and Derived Performance Metrics — RBF Neural Network Classifier

**Confusion Matrix — RBF Neural Network (5-fold CV, n=190)**			
	**Predicted by model**		
**Actual diagnosis**	**Cirrhosis (Negative)**	**HCC (Positive)**	**Row total**
**Cirrhosis (n=132)**	**TN = 128** *(True Negative)*	**FP = 4** *(False Positive)*	132
**HCC (n=58)**	**FN = 1** *(False Negative)*	**TP = 57** *(True Positive)*	58
**Column total**	129	61	190

Derived performance metrics:

**Metric**	**Value**	**Formula (typical values)**
Sensitivity (Recall)	**98.28%**	*TP / (TP + FN) = 57 / 58*[2]
Specificity	**96.97%**	*TN / (TN + FP) = 128 / 132*[2]
PPV (Precision)	**93.4%**	*TP / (TP + FP) = 57 / 61*
NPV	**99.2%**	*TN / (TN + FN) = 128 / 129*
AUROC	**0.991**	*DeLong method (MedCalc)*
95% CI AUROC	**0.986–0.995**	*DeLong method (MedCalc)*
95% Bootstrap CI	**0.987 to 0.994**	*BCa bootstrap, 1000 iterations*
Youden Index J	**0.9555**	*(Sensitivity + Specificity – 1)*
Confusion value	**0.03**	*Misclassified / Total*

Table S2 Calibration Results from 4 Neural Network Models tested

Neural Network Architecture Calibration	Slope	Intercept	Brier Score	Interpretation
Radial Basis Function	0.991	0.027	0.0131	Excellent calibration. Slope ≈ 1 indicates near-perfect alignment between predicted and true probabilities. Slight positive intercept suggests a minimal upward bias. Low Brier score reflects highly accurate probability estimates.
Logistic	0.969	0.037	0.0224	Good calibration. Mild overconfidence (slope < 1) and small positive intercept indicate slightly inflated probabilities. Brier score is higher than RBF but still strong.
Support Vector machine	1.1	-0.12	0.032	Underconfident predictions (slope > 1) and negative intercept indicate systematically low probability estimates. Highest Brier score shows weakest probability accuracy. Typical for SVMs due to non-native probability outputs.
Random Forest	1.078	-0.019	0.0062	Best Brier score, but slope > 1 indicates probability compression (underconfidence). Slight negative intercept suggests probabilities are slightly too low. Common behaviour for tree ensembles.

Table S3 Comparison of performance between different neural network architectures

Model Comparison	McNemar p-value	Interpretation
RBF vs LOGISTIC	0.004	Significant difference. These models disagree on a meaningful number of samples; their decision boundaries are not equivalent.
RBF vs SVM	0.002	Significant difference. RBF and SVM make different mistakes on the same cases.
RBF vs RANDOMFOREST	0.004	Significant difference. Random Forest predictions diverge strongly from RBF.
LOGISTIC vs SVM	0.917	No significant difference. Logistic and SVM behave almost identically; they make similar errors.
LOGISTIC vs RANDOMFOREST	0.000	Highly significant difference. Random Forest disagrees with Logistic on many samples.
SVM vs RANDOMFOREST	0.000	Highly significant difference. Random Forest also disagrees strongly with SVM.

Machine-Learning Models tested in this work

Four machine-learning models were used to generate diagnostic class probabilities from patient measurements. They were calibrated and compared with other – resulting in the choice of the Radial Basis Function Neural Network for data analysis. A brief description of each model is presented.

Radial Basis Function Network (RBF)

The Radial Basis Function network is a neural architecture designed to recognise complex, non-linear patterns and is applied widely to clinical sensor data. In this case it operates by measuring how closely a patient's measurements resemble representative patterns ("centres") learned during training. Each centre reflects characteristic signal behaviour associated with one of the diagnostic groups. The network combines these similarity measures to produce smooth, well-calibrated probability estimates for each class. Because RBF networks respond strongly to local structure in the data, they are effective when subtle, non-linear relationships exist between variables. Model performance is evaluated using stratified k-fold cross-validation to ensure stability across different patient subsets.

Penalised Logistic Network (LOGISTIC)

The Logistic network is based on penalised logistic regression and is a widely used and clinically interpretable statistical method. In this case, it estimates the probability of disease by combining multiple patient variables, each weighted according to its contribution to the outcome. The penalty term prevents overfitting, ensuring that the model remains robust even when many predictors are included or when variables are correlated. This approach produces smooth probability estimates that are straightforward to interpret clinically and avoids overly extreme predictions. The model supports probability-based outputs and is validated using stratified k-fold cross-validation to confirm consistent diagnostic performance.

Support Vector Machine Network (SVM)

The Support Vector Machine is a margin-based classifier also widely used for clinical data applications. In this case it identifies the boundary separating diagnostic groups by focusing on the most informative patient cases. These "support vectors" define the decision surface, enabling the model to distinguish between classes even when the data are not linearly separable. Although SVMs do not naturally produce probability estimates, calibrated methods are applied to convert their decisions into clinically meaningful likelihoods. This approach is effective when the diagnostic boundary is sharp and driven by a subset of highly informative features. The model is evaluated using stratified k-fold cross-validation to ensure reliability across different patient subsets.

Random Forest Network (RANDOMFOREST)

The Random Forest model is an ensemble method that combines many decision trees to improve diagnostic reliability. Each tree is trained on a weighted subset of the data to maintain balanced representation of both classes when the dataset is imbalanced. At each decision point, the tree considers a limited number of variables, reducing overfitting and encouraging robust, clinically meaningful patterns. Splits are selected using the Gini impurity measure, which indicates how effectively a variable separates the diagnostic groups. Each terminal node stores the most likely class and an estimated probability distribution. The model averages these probability estimates across all trees to produce stable predictions. It retains each tree's variables, thresholds, and probability distributions to support transparency and reproducibility. Performance is assessed using stratified k-fold cross-validation, and outputs can be expressed as either majority-vote classifications or class probabilities.
